# Usability evaluation of the “Teen ‘n Fit” mobile health application: A formative study among Indonesian adolescent girls

**DOI:** 10.1371/journal.pone.0337013

**Published:** 2025-11-14

**Authors:** Dwi Sisca Kumala Putri, Kencana Sari, Nur Handayani Utami, Nazarina Nazarina, Tiara Amelia, Nadira Yuthie Salwa, Ning Sulistiyowati, Adindra Vickar Ega, Muhammad Azzumar, Rika Rachmawati, Salimar, Mieska Despitasari, Donny Kristanto Mulyantoro

**Affiliations:** 1 Research Center for Public Health and Nutrition, Research Organization for Health, National Research and Innovation Agency, Bogor, Indonesia; 2 Faculty of Public Health, Universitas Indonesia, Depok, Indonesia; 3 Research Center for Equipment Manufacturing Technology, Research Organization for Energy and Manufacture, National Research and Innovation Agency, Tangerang Selatan, Indonesia; 4 Research Center for Electrical Technology, Research Organization for Energy and Manufacture, National Research and Innovation Agency, Tangerang Selatan, Indonesia; 5 Research Center for Preclinical and Clinical Medicine, Research Organization for Health, National Research and Innovation Agency, Bogor, Indonesia; RAK Medical and Health Sciences University, UNITED ARAB EMIRATES

## Abstract

**Background:**

Mobile Health (mHealth) Applications offer a promising approach to promote the adoption of healthy nutrition and behavior among adolescent girls. A tailored mobile app, *Teen ‘n Fit,* was developed to support adolescent girls in Indonesia to assess their nutritional status, physical activity, and eating behavior; as a nutrition education media; and as a reminder of iron folic acid consumption. However, it is essential to measure the app’s usability prior to the release.

**Objective:**

This study aimed to measure the usability of a mobile application designed to promote nutrition and healthy behavior of adolescent girls by modifying the mHealth App Usability Questionnaire (MAUQ).

**Methods:**

A cross-sectional usability study was conducted on 64 adolescent girls aged 15–18 from a public high school in Depok, Indonesia. Participants completed app-based tasks and filled out a post-task usability questionnaire. The usability questionnaire was adapted from a validated MAUQ for a standalone mHealth app. The exploratory factor analysis was conducted to determine the items constituting each component in the modified MAUQ. Mann-Whitney analysis was employed to analyze the difference in usability score means based on participants’ characteristics.

**Results:**

The modified MAUQ demonstrated strong reliability (Cronbach’s alpha = 0.945). The app achieves a strong usability score, 6.0 ± 0.8 out of 7, with 79.7 percent of participants reporting no prior mHealth experience. The score among participants who occasionally used mHealth applications was higher (p = 0.046) than those who had never made prior use.

**Conclusion:**

The findings indicate strong usability potential of Teen ‘n Fit as a digital health promotion tool for adolescent girls; however, future efforts in conducting effectiveness tests and maintaining user engagement are needed.

## Introduction

Adolescence is a critical development period characterized by rapid physical, emotional, and behavioral changes [1]. Due to rapid development and higher nutritional needs, especially iron, adolescent girls frequently suffer from thinness, chronic undernutrition, and micronutrient deficiency. The pooled prevalence of anemia in adolescent girls varies from about 20 percent to almost 40 percent [2–4], and the prevalence of thinness in adolescent girls falls between 10 and 25 percent [5–8]. In Indonesia, 23 percent of adolescent girls suffer from anemia, and 46.6 percent of adolescent girls aged 15–19 years were at risk of chronic energy deficiency [9]. Addressing these nutritional challenges encompasses immediate benefits in adolescents’ current health, long-term advantages in adulthood, and reduced health risks for the next generation of children [10].

During adolescence, emphasizing behavior modification is particularly essential. Comprehensive nutrition improves adolescent knowledge and promotes healthy behavior [11]. The findings from a study conducted in Indonesia indicate that educational interventions focused on nutrition have a substantial impact on nutritional knowledge, physical activity habits, and the consumption of vitamin A-rich fruits and vegetables among adolescent girls [12].

Smartphone and internet usage among adolescents has surged globally [13–16], and mobile devices are increasingly leveraged as technology-based interventions to promote adolescent health [17–19]. It shows the potential of using mobile applications as a nutrition educational platform. Despite the rise of health-related apps, few are specifically designed for adolescent girls in low- and middle-income countries, and even fewer have undergone rigorous usability evaluation. An mHealth application for adolescent girls, *Teen ‘n Fit*, has been developed to address that gap. The application integrates features to support adolescent girls aged 15–18 in assessing their nutritional status, eating habits, and physical activity habits. However, before its public implementation, ensuring the app is intuitive, user-friendly, and engaging for its target users is critical. This study aimed to evaluate the usability of *Teen ‘n Fit by* adopting the Technology Adoption Model (TAM) and modifying the Mobile App Usability Questionnaire (MAUQ). TAM suggests that perceived usefulness and ease of use of a technology could shape an individual’s perspective toward it and drive their behavioral intention to adopt it [20,21]. Findings from this study are expected to inform the refinement of the app and support its potential role in adolescent health promotion.

## Materials and methods

### Development of the mHealth app

Teen ‘n Fit, an Android-based mHealth app, was developed between 2023 - 2025 by a multidisciplinary team of community nutrition researchers, health behavior specialists, and software developers. The application development followed the waterfall method, encompassing planning, design, development, testing, and refinement phases [22,23]. During the initial year, the focus was on building applications and usability tests. The next stages will focus on evaluating the app’s effectiveness in improving adolescent girls’ knowledge and awareness of nutrition and health.

### The instruments and features

The instrument adopted in the app to help adolescent girls evaluate their eating habits is the Adolescent Food Habits Checklist (AFHC) [24]. A preliminary reliability test of the AFHC was conducted via an online survey before the main study. The instrument was assigned to a separate sample of 38 adolescent girls aged 15–18, distinct from the usability study participants. The Cronbach’s alpha coefficient of the adopted AFHC is 0.672 (acceptable reliability), and no modifications were made to the AFHC’s items. The borderline reliability might be due to cultural or contextual differences in how certain items performed.

The Adolescent Food Habits Checklist (AFHC) [24] assesses the consumption of specific energy-dense foods, fruits and vegetables, and fats. Additionally, it examined the snacking behavior. The AFHC comprises a 23-item scale that measures dietary practices using a response format of “true” or “false.” Additionally, ten items had an alternative response option equivalent to ‘not applicable.’ One point is given for each response that was considered ‘healthy.’ Each response of ‘not applicable’ is considered a not-completed item. The final score was calculated by adjusting for ‘not applicable’ and missing responses using the formula: AFHC score = number of ‘healthy’ responses x (23/number of items completed) [24]. The results of the eating habit assessment in the app were categorized based on the tertile distribution of the AFHC total scores, which are classified as healthy (score ≥ 15), neutral (10–14), and unhealthy (≤9). Specifically, higher AFHC scores correspond to healthier eating behaviors, whereas lower scores indicate less healthy eating behaviors.

The physical activity habits instrument included in the app refers to WHO recommendations for physical activity among adolescents aged 10–17 years [25]. The instrument of physical activity habits was tested among the same group of 38 adolescent girls (aged 15–18 years) who participated in the AFHC reliability testing to identify physical activities frequently performed by adolescent girls. The developed physical activity instrument examined moderate and vigorous physical activity, including during physical education class, performed within the past seven days, along with its frequency and duration. In addition, recreational screen time was also examined, including activities such as watching TV, using subscription-based streaming services (Netflix, Disney, etc.), using smartphones for games and social media, and playing video games. Moderate and vigorous physical activity classification followed the Youth Compendium of Physical Activities, which defines intensity levels for common youth activities [26]. Each recorded activity was assigned an intensity level based on established metabolic equivalent (MET) values: moderate (3–6 METs) or vigorous (>6 METs). The result of the physical activity assessment in the app was either met or failed to meet WHO recommendations [25].

The nutritional status assessment feature in the application refers to the WHO growth standards for adolescents aged 10–19 years, specifically focusing on the Body Mass Index according to Age (BMI-for-Age) [27]. Adolescent girls are required to input their weight, height, and the date of data measurement. The BMI will be calculated and compared to the WHO BMI-for-age standard. The nutritional status assessment results in the app were severe thinness (BMI for Age Z-score (BAZ) <−3 SD), thinness (−3 SD ≤ BAZ < −2 SD), normal (−2 SD ≤ BAZ ≤  +1 SD), overweight (+1 SD < BAZ ≤ +2 SD), and obesity (BAZ > +2 SD).

**Fig 1:**
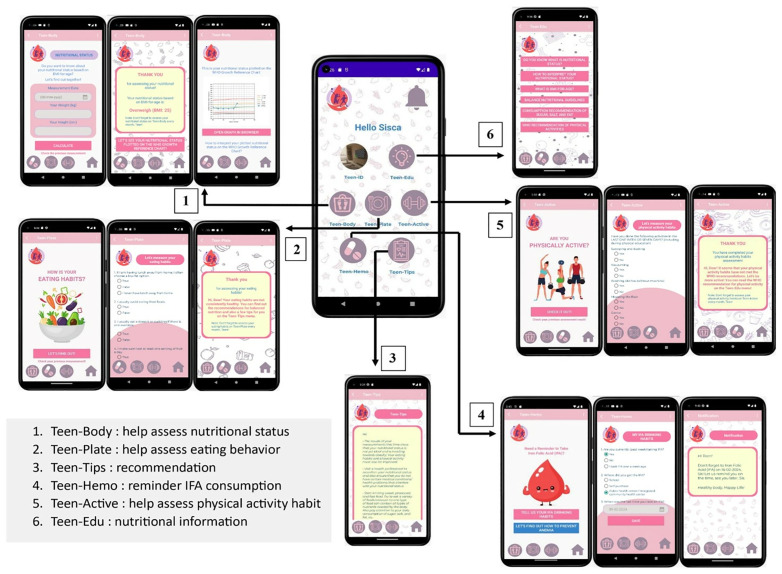
The Menus in “Teen ‘n Fit” App.

The developed app, Teen ‘n Fit, comprises six main menus (Fig 1), including Teen-Edu, which provides information and education about nutrition; Teen-Body to help calculate nutritional status and display BMI-for-Age plotted on WHO growth charts; Teen-Plate to help evaluate eating habits; Teen-Active to help assess physical activity; Teen-Hemo to record Iron Folic Acid (IFA) consumption habit and send reminders about drinking IFA; and Teen-Tips to provide recommendations based on measurements of nutrition status, dietary habits, and physical activity habits.

### Usability evaluation

This study delivers a formative usability assessment to evaluate the first usability and user perceptions of the Teen ‘n Fit mobile application before broader implementation. This usability evaluation adopts the Technology Acceptance Model (TAM) to evaluate the usability and acceptance of the *Teen ‘n Fit* app among adolescent girls because of its significant predictive value in mHealth environments, especially among adolescents, where engagement depends critically on perceived value and ease of navigation. TAM suggests that perceived usefulness (the degree to which a person believes that using a particular system would enhance their performance) and perceived ease of use (the degree to which a person believes that using a system would be free of effort) are fundamental determinants of user acceptance [28,29].

The modified Mobile App Usability Questionnaire (MAUQ) employed in this study aligns with these constructs, enabling an assessment of how these perceptions influence the intention to use the app. The perceived ease of use was assessed by items measuring the intuitiveness of the application interface and navigational simplicity. The perceived usefulness was assessed by items measuring the significance and utility of the app content (nutritional status, eating behavior, and physical activity habits assessment).

### Modification of the mobile application usability questionnaire (MAUQ)

Several validated tools have been developed to evaluate mobile app usability, including the Post-Study System Usability Questionnaire (PSSUQ) and System Usability Scale (SUS) [30]. The PSSUQ and SUS, however, were not specifically developed to assess or measure the usability of mHealth applications.

The usability evaluation of Teen ‘n Fit involved modifying a recently developed mHealth App Usability Questionnaire (MAUQ) for a standalone mHealth app developed by Zhou et al., which is highly reliable and valid to evaluate the usability of mHealth apps [31]. The MAUQ was developed by assessing the interactive mHealth app, iMHere, and the standalone mHealth app, Fitbit. The MAUQ for standalone apps originally had 18 items to be evaluated and divided into three subscales, including ease of use, interface and satisfaction, and usefulness and system information arrangement. The ease of use subscale consists of five items (S1-S5); the interface and satisfaction subscale consists of 7 items (S6-S12); and the usefulness subscale consists of 6 items (S13-S18). We modified S8 in the interface and satisfaction subscales, and S13-S15 and S17-18 in the usefulness subscale. The items mostly modified were in the usefulness subscale to correspond with the application’s function and goals (Table 1). The primary objective was to ensure that each question corresponded with the app’s functionalities and the elements of the Technology Acceptance Model (TAM) we aimed to assess (Perceived Usefulness and Perceived Ease of Use), while remaining consistent with the essence of the original items.

**Table 1:** Modification of MAUQ*.

MAUQ original version [31]	Modification	Rationale for change/modification
Ease of use		
S1. The app was easy to use.	–	
S2. It was easy for me to learn to use the. app	–	
S3. The navigation when moving between the screens was consistent.	–	
S4. The app interface allowed me to use all the functions (such as entering information, responding to reminders, and viewing information) offered by the. app	–	
S5. Whenever I made a mistake using the app, I could recover easily and quickly.	–	
Interface and satisfaction		
S6. I like the interface of the. App	–	
S7. The information in the app was well organized, so I could easily find the information I needed.	–	
S8. The app adequately acknowledged and provided information to let me know the progress of my action.	The application provides adequate acknowledgment and information for me to assess my nutritional status, eating habits, and physical activity.	Modified to reflect Teen’n Fit’s core function of enabling users to assess their nutrition, eating habits, and physical activity, as the app was not designed to monitor ongoing actions, ensuring the item remains relevant while preserving its intent of evaluating whether the app provides adequate feedback.
S9. Feel comfortable using the app in social settings.	–	
S.10 The amount of time involved in using the app has been fitting for me.	–	
S.11 I would use this app again.	–	
S.12 Overall, I am satisfied with this. app.	–	
**Usefulness**		
S13. The app would be useful for my health and well-being.	The app would be useful for helping me assess my nutritional status, eating habits, and physical activity.	Modified to specify Teen ’n Fit’s intended purpose of helping users assess their nutritional status, eating habits, and physical activity, as the app focuses on self-assessment rather than providing general health or well-being outcomes, ensuring the item accurately captures perceived usefulness within the app’s scope.
S.14 The app improved my access to healthcare services.	Removed due to inappropriateness. Changed into: The app provides helpful nutrition and health education	Changed to maintain relevance to our app’s functionality (as nutrition education tools) while aiming to preserve the intent of the original question (assessing perceived usefulness to the user’s health goals).
S.15 The app helped me manage my health effectively.	The app motivates me to maintain and monitor my health	Modified to reflect Teen’n Fit’s primary function of encouraging users to adopt and sustain healthy behaviors through motivation and self-monitoring features, rather than implying comprehensive health management, ensuring the item measures perceived usefulness within the app’s intended scope.
S.16 This app has all the functions and capabilities I expected it to have.	–	
S.17 I could use the app even when the Internet connection was poor or not available.	Removed	Removed to increase internal consistency
S.18 This mHealth app provided an acceptable way to receive health care services, such as accessing educational materials, tracking my activities, and performing self-assessment.	The app provides an easy and acceptable way for adolescent girls to perform self-assessments in calculating their nutritional status, eating habits, and physical activity	Modified to align with *Teen’n Fit*’s actual scope as a self-assessment tool for calculating nutritional status, eating habits, and physical activity, rather than implying the provision of health care services, ensuring the item accurately reflects the app’s purpose and the adolescent target population.

*Original MAUQ developed by Zhou, L et al (2019).

The MAUQ was originally in English. The modified MAUQ was translated into Bahasa Indonesia for the adolescent participants. A back-translation was performed with the assistance of a bilingual health researcher. The modified MAUQ consists of 17 items and was divided into 3 subscales: ease of use, functionality and interface satisfaction, and usefulness and system information arrangement. One item in the usefulness scale (S17) was removed to improve the reliability of the modified MAUQ. The modified MAUQ was evaluated by 35 adolescent girls, beta users of the Teen ‘n Fit App. The 35 adolescent girls were a distinct group from the participants in the usability test and the instrument reliability test. An exploratory factor analysis with varimax rotation was performed to identify the items that constitute each component in the modified MAUQ (Table 2).

**Table 2:** Exploratory Factor Analysis Results of Modified MAUQ.

MAUQ modified version (overall Cronbach’s alpha = 0.945)	Factor 1	Factor 2	Factor 3
**Ease of use aspect** **(Cronbach’s alpha = 0.887)**			
The app was easy to use	0.505	*0.644*	0.334
It was easy for me to learn to use the app	0.192	*0.845*	−0.044
The navigation when moving between the screens was consistent.	0.398	*0.582*	0.172
The app interface allowed me to use all the functions offered by the app.	0.501	*0.666*	0.333
Whenever I made a mistake using the app, I could recover easily and quickly.	0.149	*0.789*	0.270
**Usefulness and system information arrangement aspects** **(Cronbach’s alpha = 0.847)**			
The information in the app was well organized, so I could easily find the information I needed.	*0.849*	0.325	0.240
The application provides adequate acknowledgment and information for me to assess my nutritional status, eating habits, and physical activity.	*0.840*	0.436	0.152
I feel comfortable using the app in social settings.	*0.729*	0.053	0.148
The amount of time involved in using the app has been fitting for me.	*0.794*	−0.040	0.320
The app would be useful for helping me assess my nutritional status, eating habits, and physical activity.	*0.844*	0.355	0.252
The app provides helpful nutrition and health education	*0.839*	0.413	0.212
The app motivates me to maintain and monitor my health	*0.833*	0.435	0.107
The app provides an easy and acceptable way for adolescent girls to perform self-assessments in calculating their nutritional status, eating habits, and physical activity	*0.847*	0.345	0.224
**Functionality and interface Satisfaction aspects** **(Cronbach’s alpha = 0.847)**			
I like the interface of the app.	0.081	0.512	*0.570*
I would use this app again.	0.290	0.197	*0.852*
Overall, I am satisfied with this app.	0.385	0.494	*0.659*
This app has all the functions and capabilities I expected it to have.	0.223	0.027	*0.867*

The modified MAUQ demonstrated excellent internal consistency (Cronbach’s alpha = 0.945), as shown in Table 2. Exploratory factor analysis with varimax rotation revealed a three-factor structure aligning with Technology Acceptance Model (TAM) constructs. Factor 1 (Usefulness and System Information Arrangement; α = 0.847) comprised 7 items assessing perceived utility and organizational clarity. Factor 2 (Ease of Use; α = 0.887) contained 5 items measuring navigational simplicity and error recovery. Factor 3 (Functionality and Interface Satisfaction; α = 0.847) included 4 items evaluating aesthetic appeal and satisfaction. All items loaded strongly (>0.50) on their intended factors. Five original MAUQ items were modified to reflect app-specific functions (rewording usefulness items to emphasize nutritional self-assessments), and one item (original S17) was excluded to optimize scale performance.

### Usability test

Usability evaluations through field testing could provide a true portrayal of user conditions within an evolving environment [32]. This app was evaluated through field testing in a cross-sectional study. A public high school was randomly selected from 15 public schools in Depok City, West Java, for the usability test. One school was chosen due to its accessibility and the feasibility of recruiting participants within a limited timeframe. The participants in the usability test were recruited from January 2^nd^ to 10^th^, 2024. A total of 64 participants were selected using stratified random sampling from a list of eligible Grade 10 and 11 students (aged 15–18 years and owning Android devices). Stratification was based on class, with 5–6 students randomly selected from each class. Participants were instructed to complete a series of standardized in-app tasks (account setup, profile entry, BMI calculation, eating and physical activity self-assessment) designed to simulate real-life app use. These tasks were selected based on key functionalities within the *Teen ‘n Fit* app.

The participants installed the application, completed the five key tasks (Table 3), explored the application independently, and were observed through in-app logs over four days. The app logs were primarily used to confirm whether tasks were completed, not to measure timing. The in-app logs included a timestamp (date and time recorded when the user created an account or accessed the menus in the app), user ID, the result of the nutritional status assessment, and the eating and physical activity habit assessment. The number of failed attempts and time on task of all features were not recorded in the logging system. Upon concluding their task and independent exploration of the app in four days (from 15^th^ to 18^th^ January 2024), the participants were required to fill out a self-administered post-task questionnaire.

**Table 3:** The App Task Completion Rate (n = 64).

Tasks	Completion rate (%)
Account setup	100 (64/64)
Profile entry	100 (64/64)
BMI/Age Calculation	98.4 (63/64)
Eating habit assessment	98.4 (63/64)
Physical activity habit assessment	85.9 (55/64)

Table 3 showed that most app features have consistently high rates of task completion. All 64 participants (100%) completed the processes of activating their accounts and entering their profiles. Similarly, BMI/age calculations and eating habit assessments were near-universally successful, with 63 out of 64 participants completing them. However, the physical activity habit assessment task showed a noticeable decline in performance, with a completion rate of 85.9%, suggesting this specific module may require usability improvements.

The usability test examined the ease of use, functionality and interface satisfaction, usefulness and system information arrangement. Responses to the modified MAUQ items varied from a maximum score of 7, indicating strong agreement, to a minimum score of 1, indicating strong disagreement. The highest usability total score mean of the modified MAUQ was 7. The highest possible usability score was 7 (on a 7-point scale), and a few participants rated the app at this maximum on all items

We also examined gadget (smartphone and tablet) usage behavior and sociodemographic characteristics. The behavior of gadget usage includes the average time spent on smartphones/tablets per day, prior use of mHealth apps, and the number of mobile app types used. The sociodemographic characteristics include age, parents’ education, and parents’ occupation.

Participant feedback was also collected during the usability test, encompassing their favored and least preferred features, the most challenging feature to comprehend and complete, as well as suggestions for potential app development. The feedback for suggestions on further application development was obtained through open-ended questions.

### Data analysis

The gadget usage behavior, sociodemographic characteristics, and feedback from participants were summarized in percentages. The open-ended feedbacks was categorized and summarized in percentages. Each usability aspect (ease of use, functionality and interface satisfaction, usefulness, and system information arrangement) was presented in mean. Kolmogorov-Smirnov analysis was performed to test the normality of the usability average scores. The analysis showed that the distribution of the usability score data was non-normal. Mann-Whitney analysis, using IBM SPSS Statistics Version 21, was employed to analyze the difference in usability score means based on parents’ education, average time spent on smartphones/tablets per day, prior use of mHealth apps, and the number of mobile app types used.

### Ethical consideration

The Health Research Ethics Committee, National Research and Innovation Agency (No: 086/KE.03/SK/08/2023), approved the study protocol and informed consent. During recruiting, participants and parents received written information regarding the study in the form of an informed consent letter. Written parental assent and participant consent were obtained before the usability test, with the teacher serving as a witness.

## Result

### Usability test

Most of the usability test participants were 16 years old. The proportion of participants with parents who attained education at the secondary level or lower was higher (56.2%) than that of participants with parents who achieved education beyond the secondary level (43.8%). Most participants used smartphones for more than 5 hours daily, had never utilized mHealth applications, and used more than two types of mobile applications daily. The characteristics of the usability assessment participants are shown in Table 4.

**Table 4:** Usability Assessment Participants’ Characteristics.

Characteristics	n (%)
Age (years)	
• 15	26 (40.6)
• 16	31 (48.5)
• 17	7 (10.9)
Father’s Education	
• > secondary level	28 (43.8)
• ≤ secondary level	36 (56.2)
Mother’s Education	
• > secondary level	28 (43.8)
• ≤ secondary level	36 (56.2)
Average time of smartphone/tablet usage per day (hours)	
• ≤ 2	7 (10.9)
• > 2 and ≤ 5	18 (28.2)
• > 5	39 (60.9)
	Median: 7 hours
Mobile apps utilization	
• Every day	62 (96.9)
• Not every day	2 (3.1)
Previously utilized health application	
• Yes, occasionally	13 (20.3)
• Never	51 (79.7)
Number of mobile app types utilized	
• ≤ 2 types	18 (28.1)
• > 2 types	46 (71.9)

### Usability of the application

The overall usability score was 6 out of 7, suggesting very good usability for the app (Table 5). Overall, the items that received the most favorable feedback were related to functionality and interface satisfaction, as well as usefulness and system information arrangement. No significant differences were observed in the scores for ease of use, usefulness and system information arrangement, as well as functionality and interface satisfaction score, based on participants’ parental education, average time of device usage per day, and the number of mobile apps used. However, the ease of use scores among participants who had never used mHealth applications were significantly lower than those who had made prior use. The usability scores based on participant characteristics are shown in Table 5.

**Table 5:** App Usability Score based on Participants’ Characteristics.

Variables	Usability score					
	Ease of use	P-value*	Functionality and interface Satisfaction	P-value*	Usefulness and system information arrangement	P-value*
	Mean (SD)		Mean (SD)		Mean (SD)	
Father’s Education						
• > secondary level	6.0 (0.9)	0.114	6.0 (0.8)	0.738	6.1 (0.5)	0.670
• ≤ secondary level	5.8 (0.9)		6.1 (0.9)		5.9 (0.9)	
Mother’s Education						
• > secondary level	6.0 (0.9)	0.282	6.0 (0.8)	0.723	6.0 (0.6)	0.645
• ≤ secondary level	5.8 (0.9)		6.1 (0.9)		5.9 (0.9)	
Average time of smartphone/tablet usage per day (hours)						
• > 7 hours	5.8 (1.1)	0.501	5.9 (1.1)	0.263	5.9 (0.9)	0.235
• ≤ 7 hours	6.0 (0.7)		6.2 (0.5)		6.1 (0.5)	
Prior mHealth application usage						
• Yes, occasionally	6.0 (0.9)	0.046**	6.1 (0.6)	0.824	6.0 (0.6)	0.406
• Never	5.5 (1.1)	r = 0.25	6.0 (0.9)		6.0 (0.8)	
Number of mobile app types used						
• > 2 types	5.9 (0.8)	0.844	6.1 (0.7)	0.982	6.1 (0.5)	0.156
• ≤ 2 types	5.8 (1.2)		6.0 (1.3)		5.7 (1.1)	
Mean scores of each aspect	5.9 (0.9)		6.1 (0.9)		6.0 (0.8)	
The summary of the usability score means	6.0 (0.8)					

*Mann-Whitney Test.

**significant p-value ≤ 0.05.

Quantitative user feedback reveals a largely favorable user experience with practical suggestions for improvement (Table 6). Registration was the main source of confusion (46.9%), whereas one-third of participants (32.8%) reported no trouble. Users most often praised the Teen-Body module (25.0%) and the app’s visual design (21.9%), and the vast majority reported no least-liked component (81.3%). Over 40% of users perceive the application to be adequate and functional. Requested improvements converged on incorporating several features, including daily meal plan and calorie intake counter features, additional health information/education, incorporating interactive features, and small User Interface and User Experience improvements (color palette and visual clarity).

**Table 6:** Participants’ Feedback.

Feedback	n	%
Most perplexing and challenging features to comprehend.		
• None	21	32.8
• Account registration	30	46.9
• Workflow	4	6.2
• Teen-Active Menu (measure physical activity behavior)	4	6.2
• Others	5	7.9
Least favorable features.		
• None	53	82.8
• Design (Color palette, fonts, etc.)	4	6.3
• Teen-active Menu (measure physical activity behavior)	2	3.1
• Teen-Edu (nutrition education)	2	3.1
• Others	3	4.7
Most favorable features		
• Design (Color palette, fonts, etc.)	14	21.9
• Teen-Body (assess nutritional status)	16	25.0
• Teen-Plate (assess healthy eating behavior)	9	14.1
• Teen-Tips (recommendation)	9	14.1
• Teen-Edu (nutrition education)	4	6.3
• Teen-Active (assess physical activity behavior)	3	4.6
• Workflow	3	4.6
• Others	6	9.4
Suggestions for application improvement		
• The application is already proficient and functional	27	42.2
• Incorporate a daily meal plan and a calorie intake counter based on users’ nutritional status	10	15.6
• Incorporate additional health education/information, including mental health, reproductive health, sleep habits, etc.	16	25
• Incorporate interactive features (health consultation chatbot, sleep timer, etc.).	6	9.4
• Improve user interface and user experience (design elements, including theme, font, and color options)	5	7.8

## Discussion

Utilizing nutrition and healthy lifestyle applications can be economical and practical for delivering dietary and nutrition knowledge to the population, particularly among adolescents who frequently rely on smartphones and the internet. A systematic review of dietary, physical activity, and sedentary behavior apps for children and adolescents suggested that future app development should focus on customizing apps for specific population groups and incorporating health behavior theory [33].

The *Teen ‘n Fit* application offers several novel contributions to adolescent mHealth interventions. First, it is specifically designed for Indonesian adolescent girls aged 15–18, a crucial developmental period frequently neglected in localized mHealth solutions. The app material was created utilizing culturally relevant language, behaviors, and nutritional examples catered to Indonesian adolescents, ensuring greater resonance with the target users. The app content was created using a writing genre that is both familiar and appropriate for Indonesian adolescent girls. Educational materials were written in conversational Bahasa Indonesia and English, using age-appropriate vocabulary and expressions frequently used in teen communication. Healthy behavior recommendations were contextualized within school activities or daily routines. For example, reminders to consume iron-folic acid supplements were aligned with the local school-based program schedule to support current governmental initiatives.

Second, the study used a modified version of the Mobile App Usability Questionnaire (MAUQ), adapted to reflect the specific functions and educational goals of *Teen ‘n Fit*. This modification provides a closer connection between assessment measures and the health promotion objectives of the app, therefore addressing a frequent gap in the field where generic usability methods might not be able to capture context-specific functionality. Furthermore, the functions, including the nutritional status assessment based on WHO BMI-for-age criteria and iron–folic acid supplementation reminders, provide a complete health engagement platform catered to the needs and health priorities of Indonesian adolescent girls.

Compared to existing global and regional mHealth applications targeting adolescents, *Teen ‘n Fit* addresses a substantial gap. Many international apps, such as *Fitbit*, primarily focus on general fitness tracking and require high digital literacy, limiting their accessibility for younger users in low- and middle-income countries. Although apps like *NutriHealth* in Indonesia provide nutrition tracking, the app lacks integrated educational components and physical activity assessment features tailored to adolescent girls [34]. *Teen ‘n Fit* bridges this gap by offering a holistic, behavior-change-oriented platform that simultaneously educates, assesses, and motivates users within a culturally sensitive framework. Teen ‘n Fit offers a novel strategy better adapted to encourage sustainable healthy behaviors among adolescent girls in Indonesia and potentially in similar middle-income nation environments by integrating localized content, gender-specific design, and theory-driven usability evaluation.

The Teen ‘n Fit app undertook a usability test on adolescent girls in public schools in urban areas. They can represent the typical conditions of adolescents in urban areas in Indonesia. Although the respondents may not fully represent adolescent girls from rural or diverse socioeconomic backgrounds, it serves as a preliminary investigation into the mobile app usability for promoting healthy behavior. Most of the participants in this study used smartphones for more than 5 hours a day, and nearly all participants used mobile applications daily. A study showed a significant association between smartphone usage and the type of residence. Smartphone usage was more prevalent in urban areas than in rural areas [35]. Another study reveals that the median duration of daily smartphone usage among urban users is around 174.9 minutes, approximately three hours. [36], lower than the findings of this study.

The Teen n Fit application achieved an overall usability score of 6 ± 0.8 out of 7, suggesting very good usability. The items that received the most favorable feedback were related to functionality and interface satisfaction (6.1 ± 0.8). This aligns with a prior evaluation of the My-Care mobile self-care application for adolescents, which reported an overall MAUQ mean score of 6.28 ± 0.55, with the highest subscale score observed for user interface & satisfaction (6.43 ± 0.58) [37]. These results highlight that an appealing interface and easily accessible functionality are key determinants of satisfaction among adolescent users.

The app obtained very good usability scores, especially for functionality and usefulness aspects, suggesting that participants were satisfied with the content and functionality. The overall usability scores for *Teen ‘n Fit* are comparable to other mHealth apps targeting adolescents. It showed a potential for the app to be an effective health promotion tool for adolescent girls. Usability assessments of youth-targeted apps in developing countries often encounter challenges with navigation and comprehension [38], leading to moderate ratings. In contrast, *Teen ‘n Fit*’s very good scores, particularly for functionality, interface satisfaction, and usefulness, indicate that localized design strategies and culturally tailored educational content successfully addressed these common barriers.

From the perspective of TAM, the findings of this study show that Teen ‘n Fit is both practical and user-friendly for adolescent girls. The very good ease-of-use, functionality, and interface satisfaction scores indicate that the app is user-friendly. Considerably high scores in perceived utility suggest that users understand the app’s potential to improve their health behaviors. These findings align with other studies using TAM in mHealth environments, revealing that these elements greatly influence users’ behavioral intentions to use health technology and adopt healthy behavior [29]. Thus, the favorable reception of Teen ‘n Fit among the target audience emphasizes the app’s possibility for general acceptance and continuous usage.

Participants who had previously used mHealth applications reported statistically significantly higher ease-of-use scores than those without prior experience (p = 0.049). However, the small effect size indicates that previous experience with mHealth had a minimal influence on users’ perceptions of the Teen ‘n Fit’s usability. In practical terms, this indicates that individuals who had previously utilized mHealth may have experienced increased ease of use or perceived greater utility from the app; however, even those who had never engaged with mHealth could still utilize it and receive benefits. Additionally, this interesting trend warrants further study or confirmation with larger samples. Future research should include more participants with prior mHealth app experience to increase statistical power and confirm whether the small observed effect persists.

According to the Technology Acceptance Model (TAM), prior experience with similar technologies, level of digital confidence, and perceived technological competency are key determinants that influence an individual’s perception of ease of use [28]. For first-time mHealth users, limited familiarity with digital health engagement patterns may lead them to perceive applications as complex, even when interacting with objectively user-friendly designs. This emphasizes the need to include onboarding tools, such as guided lessons or assistance wizards, to assist new users and improve the first interaction.

The demonstrated usability of Teen ‘n Fit highlights its scalability as a tool for public health systems addressing adolescent nutrition and anemia in Indonesia or similar LMIC contexts. The strong app usability score in urban settings suggests potential for adoption in public health campaigns targeting anemia prevention, nutrition education, and physical activity promotion among adolescent girls. Utilizing its capabilities for iron-folic acid (IFA) adherence tracking and nutrition education, ministries of health and education could incorporate this evidence-based mHealth platform into existing school health initiatives, such as Indonesia’s Usaha Kesehatan Sekolah (UKS), to maximize impact. However, urban-rural differences in digital access consider infrastructure improvements (e.g., subsidized mobile data for health apps) and digital literacy training for adolescents.

The limitations of this study include the involvement of adolescent girls from a single public high school in an urban area and a relatively small sample size. The homogeneity of our sample limits the external validity of our findings. This may limit the generalizability of the findings, particularly for adolescents in rural settings or those with diverse sociodemographic backgrounds. The result should be interpreted with caution when extrapolating to broader populations. However, this was a formative usability study designed to gather preliminary insights before broader implementation, and recruitment was constrained by the availability of eligible participants within one school and a limited timeframe. This study aimed to pilot the mobile app in a controlled setting, focusing on usability before scaling it up to a broader population. This study could provide critical insights into the usability of mHealth interventions for adolescent girls, which can be applied to future designs and interventions. Future studies should include participants from diverse geographic locations, including rural areas, to enhance the external validity of the findings.

We modified the MAUQ, a validated usability instrument, to better suit our setting and to better fit Indonesian youth (a context-specific tool). We kept the main structure and ideas of the questionnaire, but the altered version has not been validated independently yet. This indicates that, although it presumably maintained reliability (our sample exhibited significant internal consistency for the questionnaire), the content validity may have been altered, and the adaptation may limit the comparability of our usability scores. The comparisons of the usability scores to other research utilizing the conventional MAUQ should be approached with caution, and the construct validity of the modified MAUQ needs further validation.

Another limitation of this study is that all data were self-reported by participants. However, we took steps to mitigate potential biases. We assured participants that their answers were anonymous and that there were no “right” or “wrong” responses, an approach known to improve honesty by reducing social pressure. Moreover, the use of self-administered questionnaires, with no researcher present during completion, helped reduce the interviewer’s influence on participants’ responses, allowing them to report their experiences more freely.

Our next research phase is to examine user engagement and to evaluate whether the app utilization leads to improved nutrition and health knowledge, attitudes, and behavior of adolescent girls. An effectiveness test with experimental design in a broader population will be conducted (a randomized controlled trial or a longer pre-post study) to formally test the app’s impact on adolescent nutrition knowledge and behaviors. Additionally, future studies or future versions of our evaluation should incorporate more fine-grained usability metrics, such as task completion time, number of errors or assists needed, and navigation patterns, to provide a deeper understanding of the user experience.

Another future challenge is examining users’ engagement over a longer period. Sustaining engagement in adolescent mHealth apps can be difficult. To address this, future iterations should incorporate features that enhance interactivity, such as linking the app and social media platforms. This will enable users to engage in synchronous competition with one another [39]. It is also essential to involve gamification [40] in the app, especially in the nutritional education feature/menu, to boost user motivation and learning

## Conclusion

The developed application exhibits a notable usability score, particularly regarding the functionality, user interface satisfaction, usefulness, and the arrangement of system information. Thus, it demonstrates significant potential to serve as an effective health promotion tool for adolescent girls. The future challenges include conducting the effectiveness test of the app in promoting nutrition and healthy behavior, and sustaining user engagement with the expectation that it will positively impact the adolescents’ knowledge and behavior toward nutrition and health.

## Supporting information

S1. File The Modified MAUQ. (PDF)
