# Supplementary material for: Usability evaluation of the “Teen ‘n Fit” mobile health application: A formative study among Indonesian adolescent girls
Source: PLoS One. 2025 Nov 14;20(11):e0337013. doi: 10.1371/journal.pone.0337013 (PMC12617895; doi:10.1371/journal.pone.0337013)
Supplement: S1. File — (PDF) [file pone.0337013.s001.pdf]

THE MODIFIED MAUQ

	Strongly disagree	Disagree	Somewhat Disagree	Neutral	Somewhat Agree	Agree	Strongly agree
**Ease of use aspect**							
The app was easy to use							
It was easy for me to learn to use the app							
The navigation when moving between the screens was consistent.							
The app interface allowed me to use all the functions offered by the app.							
Whenever I made a mistake using the app, I could recover easily and quickly.							
**Usefulness and system information arrangement aspects**							
The information in the app was well organized, so I could easily find the information I needed.							
The application provides adequate acknowledgment and information for me to assess my nutritional status, eating habits, and physical activity.							
I feel comfortable using the app in social settings.							
The amount of time involved in using the app has been fitting for me.							
The app would be useful for helping me assess my nutritional status, eating habits, and physical activity.							
The app provides helpful nutrition and health education							
The app motivates me to maintain and monitor my health							
The app provides an easy and acceptable way for adolescent girls to perform self-assessments in calculating their nutritional status, eating habits, and physical activity							
**Functionality and interface Satisfaction aspects**							
I like the interface of the App.							
I would use this app again.							

	**Strongly disagree**	**Disagree**	**Somewhat Disagree**	**Neutral**	**Somewhat Agree**	**Agree**	**Strongly agree**
Overall, I am satisfied with this app.							
This app has all the functions and capabilities I expected it to have.							
